# Draft genome sequence of *Leptobacillium coffeanum* (Cordycipitaceae, Hypocreales), a freshwater fungus isolated from Bohol, Philippines

**DOI:** 10.1128/mra.00138-25

**Published:** 2025-07-09

**Authors:** Julia Theresa Regalado, Ma. Carmel Javier, Jasmine Velo, Jan Felnesh Exe Bagacay, Lilcah Angelique D. Opiña, Ric Ryan Regalado, Thomas Angelo Lopez, Victor Marco Emmanuel N. Ferriols, Mark S. Calabon

**Affiliations:** 1 Philippine Genome Center Visayas, University of the Philippines Visayas 54730, Miagao, Iloilo, Philippines; 2 Division of Biological Sciences, College of Arts and Sciences, University of the Philippines Visayas 54730, Miagao, Iloilo, Philippines; 3 Graduate School, University of the Philippines Visayas, Miagao, Iloilo, Philippines; 4 University of the Philippines Diliman 54727, Quezon City, Philippines; 5 Institute of Aquaculture, College of Fisheries and Ocean Sciences, University of the Philippines Visayas, Miagao, Iloilo, Philippines; University of California Riverside, Riverside, California, USA

**Keywords:** aquatic fungi, mycology, freshwater fungi, Philippine mycology, Philippine biodiversity, fungal biosystematics, tropical fungi, hyphomycetes, *Sordariomycetes*, *Ascomycota*

## Abstract

*Leptobacillium*, a lignicolous hyphomycetous fungus composed of 11 known species, is recognized for its potential as a source of biocontrol agents and bioactive compounds. In this study, we report the draft genome of *Leptobacillium coffeanum*, a species previously isolated in Brazil and now recorded in the Philippines. This genomic resource provides a foundation for future investigations into its ecological roles, metabolic capabilities, and potential applications in agriculture and biotechnology.

## ANNOUNCEMENT

*Cordycipitaceae*, a family of fungi in the order *Hypocreales*, comprises over 1,300 species (1) with significant ecological and economic roles, including biocontrol and bioactive compound production (2). *Leptobacillium*, a genus reassigned from *Simplicillium* (Hypocreales, Hypocreomycetidae, Sordariomycetes) (3), was first introduced by Zare and Gams in 2016 during their revision of four verticillium-like species that produce whitish colonies and erect conidiophores. The asexual form of this genus is hyphomycetous, characterized by conidiophores that are mainly long, solitary phialides and show occasional irregular branching. The genus name refers to the characteristically narrow microconidia, with *Leptobacillium leptobactrum* designated as the type species (4). *Leptobacillium* is distinguished by its production of globose, slimy heads and overlapping chains of short-ellipsoidal to subglobose or obclavate conidia and is characterized by white, grayish, or cream-colored and woolly colonies on potato dextrose agar (PDA) (5). Currently, 11 *Leptobacillium* spp. have been recorded based on Index Fungorum, namely, *Leptobacillium cavernicola*, *Leptobacillium chinense*, *Leptobacillium coffeanum*, *Leptobacillium filiforme*, *Leptobacillium latisporum*, *Leptobacillium leptobractum*, *Leptobacillium longiphialidum*, *Leptobacillium marksiae*, *Leptobacillium muralicola*, *Leptobacillium symbioticum*, and *Leptobacillium xianyushanense* (6), isolated from a wide range of host and substrate diversity, including insects, fungi, plants, fresh water, murals, and rocks (7). Here, we present the draft genome of *L. coffeanum*, a species described to have white colonies, moderate aerial mycelium, spindle-shaped conidia, and phialides produced on prostrate aerial hyphae (8) isolated from submerged decaying wood in a freshwater habitat in Loon, Bohol, Philippines. This genome could serve as a valuable resource for future research and potential applications in agriculture and biotechnology. The internal transcribed spacer (ITS) region was amplified using ITS5 and ITS4 primers (9). The obtained DNA sequence (GenBank accession number PV468734) was analyzed using BLASTn (https://blast.ncbi.nlm.nih.gov/Blast.cgi) to compare against sequences in the GenBank database, showing 96.19% and 95.45% identity to *L. symbioticum* NBRC 113865 and *L. filiforme* URM 7918, respectively. The sequence was aligned with ITS sequences of *Leptobacillium* and related taxa using MAFFT v.7 (http://mafft.cbrc.jp/alignment/server) (10). Phylogenetic analysis using the maximum-likelihood method placed the sequence in a clade with *L. coffeanum*, although it formed a distinct lineage.

Submerged decayed wood samples were collected from small streams of a freshwater spring (Danicop-Ticugan) in Loon, Bohol, Philippines. The collected wood samples were subsequently incubated at 27°C for 2 weeks to allow fungal growth before isolation. Fungal fruiting bodies were observed using a stereomicroscope, and microscopic characteristics were documented using the Olympus BX53 microscope. Following the methods of Senanayake et al. 2020 (11), single spore isolation was done using PDA media. Fungal mycelia were purified using the same media and extracted using the Qiagen DNeasy Blood and Tissue Extraction Kit (12) based on the manufacturer’s protocol. The quality of the genomic DNA was subsequently checked using gel electrophoresis, Multiskan SkyHigh Spectrophotometer, and Qubit Fluorometer. Library construction was carried out using 100 ng of the genomic DNA following the Illumina DNA Prep Library Kit manufacturer’s protocol (13). The quality of the library was checked using the Agilent Bioanalyzer 2100 (Agilent, Santa Clara, CA) and Qubit Fluorometer and sequenced in 2 × 151 bp paired-end reads on an Illumina NextSeq 1000 at the Philippine Genome Center Visayas, University of the Philippines Visayas, Miagao, Philippines.

The quality of the generated sequences was checked using FastQC v.0.12.1 (https://www.bioinformatics.babraham.ac.uk/projects/fastqc/) (14) and trimmed using Trimmomatic v.0.36 (http://gensoft.pasteur.fr/docs/Trimmomatic/0.36) (15) using --phred 33 SLIDINGWINDOW:4:20 LEADING:20 TRAILING:20 MINLEN:36. Reads were processed to maintain a PHRED33 quality score and trim ends with a quality score below 20. Paired-end reads were assembled with SPAdes v.4.0.0 using --careful and -k 21,33,55,77,99,127 (16), exported into FASTA format using SeqKit v.2.8.2 (https://github.com/shenwei356/seqkit/releases/tag/v2.8.2) (17) and run through RepeatMasker v.4.1.7 (https://github.com/Dfam-consortium/RepeatMasker) (18). Minimap2 v.2.28 (19) was used to calculate the sequencing depth and genome coverage, and mapping statistics were generated using Samtools v.1.21 (20). Assembly quality was evaluated with QUAST v.5.2.0 (https://github.com/ablab/quast/releases/tag/quast_5.2.0) (21), and completeness was assessed using BUSCO v.5.7.1 (22) with the fungi_odb10 database. Default parameters were used for all tools unless otherwise specified.

The whole genome of *Leptobacillium coffeanum* assembled using SPAdes has a total number of 135 contiguous sequences, with a total length of 28,907,456 bp, an N50 size of 892,306 bp, G + C content of 50.36%, and 98.3% complete BUSCOs (Table 1). Based on these results, the whole genome assembly exhibited high quality, as evidenced by the genome size, BUSCO completeness, indicating a near-complete representation, and the majority (>96%) of sequencing reads mapped back to the assembly, with low error rates and minimal ambiguous regions. These findings provide a foundation for further investigation into the mechanisms and significance underlying these fungi and their diverse metabolites that are potential sources of bioactive and biocontrol agents, given that all the reads and whole genome assembly files were deposited and made available to the public at the National Center for Biotechnology Information.

**TABLE 1:** *Leptobacillium coffeanum* genome information and assembly quality using QUAST

Genome assembly statistics	Genome assembly
Total length (bp)	28,907,456
Number of contigs	135
Largest contig	2,917,615
N50 (bp)	892,306
N90	239,524
L50	10
L90	33
GC (%)	50.36
# N’s per 100 kbp	0.00
Complete BUSCOs (%)	98.3
Complete and single-copy BUSCOs (%)	745
Complete and duplicated BUSCOs (%)	6
Fragmented BUSCOs (%)	0
Missing BUSCOs(%)	13
Total BUSCO groups searched	758
Genome coverage	100

## Data Availability

The whole genome shotgun project was deposited in the National Center for Biotechnology Information (NCBI) under BioProject PRJNA1217502 and BioSample SAMN46493321, accession number JBLYVL000000000. The raw sequencing reads were deposited under accession number SRR33330895. The sequences for the ITS region were deposited in the NCBI under accession number PV468734.
